# Pain-related stigma as a social determinant of health in diverse pediatric pain populations

**DOI:** 10.3389/fpain.2022.1020287

**Published:** 2022-11-14

**Authors:** Emily O. Wakefield, Ama Kissi, Siddika S. Mulchan, Sarah Nelson, Sarah R. Martin

**Affiliations:** ^1^ Division of Pain and Palliative Medicine, Connecticut Children’s Medical Center, Hartford, CT, United States; ^2^ Department of Pediatrics, University of Connecticut School of Medicine, Farmington, CT, United States; ^3^ Department of Experimental-Clinical and Health Psychology, Ghent University, Ghent, Belgium; ^4^ Center for Cancer and Blood Disorders, Connecticut Children’s Medical Center, Hartford, CT, United States; ^5^ Department of Anesthesiology, Critical Care, and Pain Medicine, Boston Children’s Hospital, Boston, MA, United States; ^6^ Department of Psychiatry, Harvard Medical School, Boston, MA, United States; ^7^ Department of Anesthesiology & Perioperative Care, Center on Stress & Health, University of California Irvine School of Medicine, Irvine, CA, United States

**Keywords:** pediatric pain, stigma, social determinant of health, intersectional stigma, health inequity

## Abstract

Pediatric patients with invisible symptomology, such as chronic pain syndromes, are more likely to experience pain-related stigma and associated discrimination by others, including medical providers, peers, school personnel, and family members. The degree of this pain-related stigma may depend on several social dimensions, including observer (e.g., attentional and implicit biases) and patient characteristics (e.g., racial identity, socioeconomic stressors). In this mini-review, we introduce the concept of pain-related stigma, and the intersectionality of stigma, within the context of social determinants of health in pediatric pain populations. Stigma theory, observer attentional biases, healthcare provider implicit/explicit biases, adverse childhood experience, and psychophysiology of socio-environmental stressors are integrated. Several ethical, clinical, and research implications are also discussed. Because the study of pain-related stigma in pediatric pain is in its infancy, the purpose of this conceptual review is to raise awareness of the nuances surrounding this social construct, propose avenues through which stigma may contribute to health inequities, present frameworks to advance the study of this topic, and identify areas for further investigation.

## Introduction

According to the International Association for the Study of Pain (IASP), pain is an “unpleasant sensory and emotional experience associated with, or resembling that associated with, actual or potential tissue damage” (1). It is common for individuals experiencing ongoing pain to seek medical treatment and support from others in their lives who will aid in their recovery. However, research has suggested that not all of these individuals may receive the same level of support from others, or even worse, some may be subjected to stigmatization and devaluation based on their pain and unable to access appropriate care (2, 3). Stigma is a socio-politically constructed concept that occurs when individuals are discredited, judged, and criticized for possessing an attribute considered “spoiled” by society, demoting their power or status (4). Stigma exists across interpersonal, organizational, community, and public policy domains (5) and often leads to stereotyping, exclusion, and/or ostracizing an individual. Pain-related stigma occurs when an individual is devalued based on their pain condition (6). In adults with chronic pain, stigma has been linked to various adverse health outcomes (i.e., increased stress and anxiety, social isolation, and employment difficulties) (2, 7). Pediatric pain-related stigma research is a growing field of study (6), and emerging data indicates that pediatric pain populations are not exempt from these negative social experiences (8, 9). Specifically, a recent study described that adolescents with chronic pain frequently experience pain-related stigma from others, including medical providers, school personnel, family members, and peers, and internalized stigma (10).

Stigma has been considered a fundamental cause of health inequities (11) and as such represents an important social determinant of health. Accumulating literature demonstrates the prevalence and potential negative effects of pain-related stigma (a social construct) in pediatric pain populations (10, 12–14). However, pain-related stigma has yet to be recognized or characterized as a key social determinant of health in youth with pain conditions. Recognition of pain-related stigma as a social determinant of health would encourage the evaluation and increased intervention of the impact of pain-related stigma on children or adolescents and its intersection with other stigmatized identities (i.e., chronic pain and person of color).

Preliminary research has put forth a conceptual framework of pain-related stigma in youth with chronic pain (6). This framework identified precipitating factors, such as diagnostic uncertainty and pain invisibility that may interfere with the support of others (i.e., medical providers, school personnel, family members, and peers) and also highlighted adverse outcomes to pain-related stigma in youth with chronic pain (6). The purpose of this mini-review is to raise awareness of pain-related stigma as a social determinant of health building upon the preliminary pain-related stigma research in youth with chronic pain, and identify social dimensions in diverse pediatric pain populations that may exacerbate stigmatization. Specifically, we will discuss implications of pain-related stigma within a socio-ecological context and the implications of intersecting stigmatized identities, observer bias, and socio-ecological stressors.

## Social dimensions of pain & observer bias contributing to pain-related stigma

Precedent factors including pain invisibility, and diagnostic uncertainty reported by adolescent chronic pain populations contribute to pain-related stigma (6, 10). Pain creates ambiguous interactions with others because it is “invisible” to observers who can only rely on the individual's report of pain, and simultaneously individuals in pain need to rely on observers believing their reports. Relatedly, diagnostic uncertainty or lack of “medical evidence” sometimes leaves youth with pain open to the interpretation that their condition is either fabricated or a purely psychological issue (10). Investigating factors that contribute to stigma responses from others is vital to the identifying targets for interventions to reduce pain-related stigma in youth. Racial and ethnic inequities in pain assessment and management have been established (15–17), and a growing body of research focuses on gender and socioeconomic inequity has emerged (18, 19); however, a dearth of pain-related stigma research exists in pediatric populations.

### Observer biases

Observer biases have been implicated as an important provider factor perpetuating pain-related stigma (6). In order to decipher pain-related stigma responses from observers toward youth with chronic pain, vignette studies have been used. These findings have suggested that the degree to which there was medical justification for youths' pain report influenced whether healthcare providers (20) and school teachers (21) provided supportive responses. In other words, less medical evidence led to less social support provided. This research is consistent with patient-reported perceptions of pain-related stigma (10). These vignette methodologies allow insight into potential explicit biases, which are more deliberately expressed attitudes or beliefs about a specific group. The role of unconscious or automatically occurring attitudes of others, known as implicit biases, as a contributor to pain-related stigma in pediatric pain is less known. However, research on implicit biases of observers may be pivotal in understanding disparities in pain care (22, 23). However, relatively few empirical studies have examined the extent to which observer biases may account for disparities in how pediatric pain is assessed and treated. In this section, recent research findings using research methods to evaluate two observer biases: attentional and implicit biases, are discussed.

#### Attentional biases in pain

Attentional processing of another's pain is viewed as a prerequisite for adequate pain care (24, 25). Corroborating this idea, prior work has indicated that observers who are more attentive to the pain of others demonstrate more accurate pain detection and caregiving behaviors (26, 27). However, research examining how the perceived gender and race/ethnicity of pain sufferers may impact observers' attentional processing of pain is virtually non-existent in pediatric populations. Adult-focused research suggests that the initial attention of adult observers is drawn more easily to pain expressed by men relative to women (28), and Black relative to White individuals (29). (Of note, the existing gender bias literature has focused on binary gender groups: men vs. women). Furthermore, evidence indicates that during later attentional phases in the Kissi et al. study, White observers with stronger false pain-related beliefs (i.e., White individuals feel pain more easily than Black individuals) tend to demonstrate less difficulty disengaging from Black relative to White pain faces (29). These results indicate that observers' attentional processing of pain: (1) may differ depending on the attentional component under scrutiny and (2) may be impacted by their pain-related beliefs.

Despite being instructive, it is important to note that the above-described studies are focused on adults in pain and may not readily inform us about how observers, including physicians, may act out on pain-related stigma beliefs for several reasons. First, images of computer-generated individuals or actors were used to represent individuals in pain which may not capture all relevant dimensions of real expressions. Second, no behavioral indices of pain care, such as assessments of pain detection (30) and inclination to prescribe analgesics (17) were integrated to examine how these relate to pain-related stigma beliefs. Third, in each of these studies, attention was measured indirectly (i.e., *via* response times) in a relatively static measure (i.e., during a single time point). In real-life contexts, however, attentional processing occurs over time (27, 31). Despite these limitations, these studies attempt to understand precedent factors related to observer biases *via* attentional bias that may contribute to the pain-related stigma experiences in individuals with pain. It is pivotal that future work examines observer attentional processing of the pain in healthcare providers, school personnel, family members, and peers toward pediatric populations.

#### Implicit and explicit biases in pain

Most of the implicit bias in pain research has been focused on healthcare provider racial/ethnic bias in adults (32, 33), which has been associated with poorer communication and less satisfaction (34), lower quality of care, and poor clinical decision-making (33, 35, 36). Emerging evidence indicates that pediatric populations may experience similar implicit biases among healthcare providers (37), but some of the research is mixed. These findings have revealed that while medical providers held implicit attitudes that Black Americans and men were more pain-tolerant than others, their decision-making did not consistently differ based on patient's race and gender (14, 22). While these findings suggest that provider bias may not routinely impact clinical decision-making, this conclusion is inconsistent with the documentation of pediatric pain patients' experiences of inequities in healthcare settings (6, 14, 22).

Beyond implicit racial/ethnic biases in healthcare providers, some evidence exists to show gender and socioeconomic biases in pain. Cohen et al. asked adult observers to rate the pain of a gender-ambiguous child in a video who the researchers randomly assigned a gender. The findings demonstrated that observers rated boys' pain higher than that of girls, and the adult participants reported explicit beliefs that girls are more sensitive to pain; thus, boys' pain was taken more seriously (38). These findings were partially replicated by Earp et al., who also found that observers rated boys' pain higher than girls', but no explicit bias beliefs were reported (19). Observer bias research has yet to evaluate implicit bias in young gender-diverse populations (i.e., gender fluid, transgender) who likely experience this pain-related stigma. Similar to gender bias research, a recent study showed that adult observers perceived children with low socioeconomic status as less pain sensitive (18). Observer bias in gender and socioeconomic status highlights the necessity to study factors contributing to pain-related stigma in diverse pain populations. Future research should also focus on delineating sources of bias from specific support systems, specifically family members, school personnel, and peers.

## Psychophysiology of socio-environmental stressors in pediatric pain

Stigma research has largely focused on the individuals experiencing stigma, those who perpetuate stigma, or both. It is critical that pain-related stigma is viewed within a social-environmental context (5). Psychosocial stress associated with socio-environmental factors that are can occur at the individual, family, or community level. Extensive evidence from broader literature links the experience of social stressors (e.g., social isolation, negative social interactions) to poor indices of health (11, 39–41), and underscores the mediating effect of biological stress responses and the physiological consequences of stress. As described above, pain-related stigma and the associated biases, discrimination, and treatment disparities introduce significant psychosocial stress and create barriers to appropriate care. Collectively, these experiences can prolong exposure to the social stressors shown to be associated with morbidity and mortality in pediatric and adult populations (42–45).

It has been hypothesized that social stress affects health by altering key biological systems and physiological processes implicated in disease risk (43, 46). Specifically, a variety of socio-ecological factors, including economic stability, education, access to healthcare, social support, and neighborhood environment have been shown to individually and/or collectively influence cardiovascular, neuroendocrine, immune function, and autonomic, inflammatory, and hypothalamic-pituitary-adrenocortical (HPA) axis stress response processes (40, 47–53).

Similar associations have been reported between stigma-related social stressors. Stigma, perceived discrimination, social isolation, and perceived social threat are implicated in altered biological processes including heightened sympathetic nervous system reactivity, HPA axis dysregulation, and immune dysregulation (39, 54). Stigma also significantly shapes social interactions and limits access to social support, an important social resource that can protect against the development of maladaptive physiological responses in the context of stress (43, 46, 55, 56).

The physiological impact of stigma and social stressors may be especially salient during adolescence and in youth with pain. Socialization plays an important role in adolescent development and overall well-being (57–60). The adolescent period is marked by changes in neurobiological development and may represent a critical period in the development of autonomic stress responses (61–63). Research has demonstrated that social pain and physical pain share neurobiological substrates (64, 65) and individuals with chronic pain and pain-related conditions (e.g., sickle cell disease) have been shown to exhibit autonomic nervous system dysfunction (66–69), which suggests an added vulnerability for youth with pain who are also experiencing social stress. Although limited data to date exist on the specific effects of stigma on the development of maladaptive physiological responses in pediatric populations, studies examining the effects of discrimination indicate a positive association between exposure to discrimination and heightened physiological stress responses in adolescent and young adult samples (70–73). Results from this growing body of research suggest that exposure to chronic social stressors during adolescence, including stigma and discrimination, may have a significant effect on physiological systems into adulthood (39, 73).

The connection between psychological stress and health-related outcomes in pediatric populations is also implicated in the conceptualization of toxic stress, which is defined as frequent or prolonged exposure to significantly stressful experiences in the absence of adequate coping and resilience factors (e.g., supportive relationships) (74, 75). Research to-date has focused on several aspects or drivers of toxic stress, including adverse childhood experiences (ACEs; abuse/neglect, parent/guardian separation or divorce, etc.), which has been a strong recent focus of research in pediatric pain populations. For example, evidence suggests that youth with chronic pain report exposure to ACEs at a disparately higher rate when compared to non-pain peers (76) and the average population (77), which may then compound those youths' risk for poorer physical and mental health long-term. Outside of external sources of stress such as ACEs, it has also been recently proposed that pediatric pain may be a source of toxic stress in and of itself, due to its added demand on external coping resources and protective relationships and potential for pain-related stigma and healthcare uncertainty (78). Indeed, stigma and racial/ethnic discrimination have been identified as sources of toxic stress in other adult and pediatric populations with evidence suggesting that the stress imposed by these experiences is significantly associated with poorer psychological and health-related outcomes (79, 80). Relatedly, as mentioned above, youth with chronic pain and in general, racialized and other marginalized groups (e.g., women) frequently experience pain-related stigma due to unfounded beliefs about medication seeking in acute care settings (11, 81), which we propose likely adds significant stress and over taxation on an already sensitive nervous system. In other health populations, stigma has been found to frequently co-occur with adverse childhood experiences and significant psychological stress (82, 83). However, these relations remain poorly understood in pediatric chronic pain populations.

In terms of treatment, burgeoning research has suggested that youth with a history of stressful or traumatic experiences may not respond as robustly to traditional chronic pain therapies (e.g., cognitive-behavioral therapy) (77, 84, 85). In parallel, research shows that individuals who experience stigma or (relatedly) mistrust in healthcare settings may not as readily engage in needed healthcare, which could put them at greater risk for poorer health-related outcomes (11, 86). It may be that the experience of pain-related stigma compounds the inherent stress associated with seeking out necessary care for pain, especially in marginalized groups. Consideration of the underlying neurobiology of stress in the context of chronic pain and pain-related stigma is also warranted as the interacting effects of these multifaceted neurophysiological processes may further exacerbate poor outcomes and disease risk. Although cognitive-behavioral, neuromodulatory, and biofeedback interventions have been cited as promising treatment approaches to address physiological stress responses in pain populations (87–90), evidence on how to best address and reduce systemic treatment barriers and inequities associated stemming from pain-related stigma and associated biases is lacking. Future research needs to examine associations among pain-related stigma, physiological stress responses, and health outcomes in diverse pediatric pain populations through a trauma-informed lens to increase the efficacy and inclusive nature of pain evaluation and treatment.

## Discussion

Pain-related stigma in diverse pediatric pain populations is an emerging field of study that requires multifaceted approaches to investigation. Namely, research that considers stigma as a social determinant of health will be vital to improve the health equity and wellness for this young population. Preliminary findings indicate that pain-related stigma is experienced by youth with pain conditions (10, 91), which is even more challenging for individuals from racialized groups (16). We also demonstrated that this social phenomenon in combination with intersecting social-environmental factors have a clear, but understudied, adverse physiological impact. Thus, greater understanding in this area through systematic and broad-based research in a variety of populations is crucial to moving the field forward. In this mini-review, we presented several individual, interpersonal, and systemic domains of this social determinant of health in order to guide future research in this area.

The substantive areas which we identified include pain-related stigma in populations with intersecting stigma identities, the importance of understanding observer biases, and possible neurophysiological impacts of social-environmental stress. In each area, there are important research and clinical implications for future practice. To begin, research on pain-related stigma in young individuals is limited mostly to qualitative research (6, 10, 92) and White, non-Hispanic samples. More availability of validated measures is needed to evaluate the different types of pain-related stigma (i.e., felt, internalized, and anticipated) (10), and their associations with health outcomes, along with a focus on diverse pediatric pain populations. Next, we justified the need for more research on how observer bias (with a particular focus on attentional, explicit, and implicit bias) contributes to disparities in pain care, through social experimental and clinical research methods. However, there is a dearth of research on observer bias and its impact in pediatric pain populations. Research in marginalized groups is particularly lacking in gender diverse and low socio-economic young populations. Lastly, the development of mechanisms regarding neurophysiological impact of pain-related stigma and the additive effect of social-environmental stress is warranted.

The further development of theoretical frameworks that incorporate multilevel social dimensions (i.e., the individual, interpersonal, and systemic level) will guide research and, in turn, advance the field of pain-related stigma in diverse pediatric pain populations. Integrating work that emphasizes the role of social and healthcare provider interactions in pain-related stigma (6, 93) with other health-related stigma frameworks that incorporate broader social forces and the appreciation for intersecting stigmas could improve the study of pain-related stigma as a social determinant of health. The Health Stigma and Discrimination Framework may be one such framework as it not only takes into account socioecological factors and intersecting stigmas, but also identifies potential drivers and facilitators of stigma which may inform potential research or intervention targets (5).

Without an understanding, appreciation, and interventions to target the mechanisms and consequences of pain-related stigma and its overlap with racialization and marginalization, pain-related stigma may continue to perpetuate pain treatment inequities and health outcomes in pediatric pain. Unfortunately, there is a lack of research on the effectiveness of stigma interventions, but recent work has proposed frameworks and key strategies to address stigma and biases in healthcare settings. Collectively, this work highlights that interventions that focus on increasing one's awareness of biases without also teaching concrete strategies are not effective and may contribute to avoidance and anxiety (94, 95). Identified key stigma intervention components include (1) provision of information on stigma, the condition, and how healthcare providers can play a role in improving health outcomes, (2) skills-based training that target provider behavior and communication change, (3) inclusion of members of the stigmatized group in the intervention and modelling person-first behaviors, (4) teaching coping and behavior regulation skills, and (5) non-judgmental training environments that promote responsibility as opposed to guilt, and (6) system-level approaches to address potentially stigmatizing policies (95–98). Research also points to the need for interventions that address intersecting stigmatized identities, the evaluation of the effects of stigma interventions on patient care experiences, and the study of ongoing intervention efforts as opposed to single, brief interventions (96).

Collectively, this growing body of literature provides a promising foundation for the further study and characterization of pain-related stigma as a social determinant of health in youth with pain conditions. Importantly, the broader stigma literature also points to frameworks to inform the development of interventions aimed at addressing multilevel and multidimensional drivers of stigmatization. Without an understanding, appreciation, and interventions to target the mechanisms and consequences of pain-related stigma and its overlap with racialization and marginalization, pain-related stigma may continue to perpetuate pain assessment and treatment inequities in pediatric pain.
